# Hemoglobin response to iron folate supplementation and associated factors among pregnant women attending public hospitals in Addis Ababa, Ethiopia: a longitudinal quasi-experimental study

**DOI:** 10.3389/fpubh.2025.1569643

**Published:** 2025-09-24

**Authors:** Zeleke Endalew Admass, Abraham Dessie Gessesse, Haimanot Andualem Ayalsew, Habtemariam Mulugeta Abate, Abebaye Aragaw Leminie, Diresibachew Haile Wondimu

**Affiliations:** ^1^Department of Biomedical Sciences, College of Health and Medical Science, Dilla University, Dilla, Ethiopia; ^2^Department of Pediatric and Child Health Nursing, College of Health Sciences, Woldia University, Woldia, Ethiopia; ^3^Department of Biomedical Sciences, College of Medicine and Health Sciences, Haramaya University, Harar, Ethiopia; ^4^Department of Biomedical Sciences, College of Medicine and Health Sciences, Wollo University, Dessie, Ethiopia; ^5^Department of Physiology, School of Medicine, College of Health Sciences, Addis Ababa University, Addis Ababa, Ethiopia

**Keywords:** anemia, hemoglobin level, iron deficiency, iron-folate supplementation, pregnancy, dietary diversity, micronutrient supplementation, maternal health

## Abstract

**Background:**

Iron-folate supplementation is a common recommended strategy for reducing the incidence of anemia in pregnant women. However, studies on the hemoglobin response to iron folate supplementation and factors associated with the effectiveness of the intervention in developing countries, including Ethiopia, are limited and requires further investigation.

**Objective:**

This study aimed to assess the hemoglobin response to iron folate supplementation and associated factors among pregnant women attending public hospitals in Addis Ababa, Ethiopia.

**Methods:**

A quasi-experimental longitudinal study was conducted in public hospitals in Addis Ababa between May 1, 2023, and March 30, 2024. A total of 410 participants were selected via systematic random sampling. The data collection methods included participant interviews, medical record reviews, laboratory tests, and anthropometric assessments. Statistical analyses were carried out via SPSS Version 27. Descriptive statistics were used to describe the profile of the study participants. A *p* value of less than 0.05 was considered statistically significant. Logistic regression analysis was performed, and adjusted odds ratios (AORs) with 95% confidence intervals (CIs) were calculated to identify significant associations.

**Results:**

A total of 59.3% of pregnant women exhibited an inadequate hemoglobin response to iron-folate supplementation, and 17% remained anemic despite supplementation. Early ANC booking (AOR = 3.9, 95% CI: 2.4–4.2), iron-folate intake for more than 2 months (AOR = 2.6, 95% CI: 1.6–4.2), adequate dietary diversity (OR = 3.4, 95% CI: 2.1–5.6), and primiparity (OR = 2.4, 95% CI: 1.4–4.2) were significantly associated with having an adequate hemoglobin response.

**Conclusion:**

Results of this study showed that majority of the pregnant women living in Addis Ababa region demonstrated an in-adequate hemoglobin response to iron-folate supplementation. Early timing of antenatal care, prolonged iron-folate intake, primiparity, and adequate dietary diversity are linked to adequate hemoglobin response. Addressing these key factors could help reduce the burden of anemia during pregnancy and improve maternal and fetal health outcomes.

## Introduction

Anemia is a hematological disorder characterized by a reduced concentration of hemoglobin or a decreased number of red blood cells, leading to impaired oxygen delivery to body tissues (1). It is a multifactorial condition that can result from deficiency of essential micronutrients like iron and folate (2). Iron deficiency anemia (IDA), the commonest form of anemia, results from inadequate iron intake, poor absorption, or increased physiological demands, particularly during pregnancy (3). Megaloblastic anemia, caused by folate or vitamin B12 deficiency, arises from impaired DNA synthesis in red blood cell precursors (4).

Anemia is a major public health issue affecting maternal and child health, particularly in low- and middle-income countries including Ethiopia. It affects approximately 38% of pregnant women worldwide, contributing to maternal morbidity and mortality, as well as adverse pregnancy outcomes such as preterm delivery and low birth weight (5). In Ethiopia, anemia remains a significant problem, with an estimated 26.4% of pregnant women affected (6).

Iron and folate supplementation during pregnancy is a widely recommended intervention to prevent and treat anemia, with proven benefits in improving maternal hemoglobin levels and reducing the risk of maternal anemia and its complications (7). Despite these known benefits, the effectiveness of iron-folate supplementation can be influenced by several factors, including the woman’s baseline hemoglobin level, compliance with supplementation, dietary practices, gastrointestinal side effects, elevation of residence and the presence of underlying health conditions such as infections or chronic diseases (8, 9). In Ethiopia, iron-folate supplementation has been part of the national antenatal care (ANC) package, with efforts to improve coverage and adherence (10). However, studies show that compliance with supplementation and the overall impact on maternal hemoglobin levels remain suboptimal, raising concerns about the adequacy of the intervention and the need to assess other contributing factors (11–13).

Given the diverse factors that influence the hemoglobin response to iron-folate supplementation, a comprehensive assessment is crucial to optimize the effectiveness of the intervention. This study aimed to assess the hemoglobin response to iron-folate supplementation and identify associated factors among pregnant women attending public hospitals in Addis Ababa, Ethiopia. By understanding the factors that affect the hemoglobin response to iron-folate supplementation during pregnancy, this study could contribute to improving maternal health outcomes and informing strategies to enhance the implementation of supplementation programs.

## Methods

### Study period and setting

The study was conducted in selected public hospitals in Addis Ababa, the capital of Ethiopia, from May 1, 2023, to March 30, 2024. Among the 12 government hospitals, three hospitals, namely, Zewditu Memorial Hospital, St. Paul’s Millennium Hospital Medical College and Menelik Hospital, were selected via a simple random sampling technique. Pregnant women attending ANC units in the aforementioned hospitals were approached. The city has a projected population of 3,603,000, and 49.97% are females, 34.4% of whom are in the reproductive age group, according to the 2019 CSA projection (14). The city lies at an elevation of 2,355 meters above sea level.

### Study design

An institution-based longitudinal quasi-experimental study design was employed from May 1, 2023, to March 30, 2024.

### Population

#### Source population

All pregnant women above 18 years of age who booked for ANC at the selected public hospitals in Addis Ababa, Ethiopia, were included.

#### Study population

All randomly selected pregnant women who were above 18 years of age had ANC booking during the time of data collection at each selected public hospital in Addis Ababa, Ethiopia.

### Eligibility criteria

#### Inclusion criteria

Women with singleton pregnancies above 18 years of age, signed the written informed consent form, started oral iron-folate supplementation with Hb levels >7 g/dL, and had ANC follow-up at the selected public hospitals during the time of data collection in Addis Ababa, Ethiopia.

#### Exclusion criteria

Women with severe anemia initially and throughout pregnancy (Hb < 7 g/dL), iron folate tablet intake prior to conception, blood transfusion, smoking, comorbid conditions, including but not limited to hypertension, Diabetes mellitus, chronic kidney disease, known hematologic disorders, and infections, including helminthiasis, HIV and malaria, were included.

### Sample size determination

The sample size was determined using single population proportion formula as follows:


$$n=\frac{z^{2}p(1-P)}{D^{2}}\;$$

Where:

- n = required sample size
- Z = Z-score corresponding to the desired confidence level (e.g., 1.96 for 95% confidence)
- p = estimated proportion of respondents with adequate hemoglobin response to iron folate supplementation (51.5% or 0.515), taken from previous research article (9)
- D = margin of error (precision) = 5%

Using the above formula, $n=\frac{z^{2}p(1-P)}{D^{2}}\;$ = $n=\frac{\left(1.96^{2})\;(0.515)(1-0.515\right)}{0.05^{2}}$ = 384; considering non response rate of 10%, the final sample size was 422.

### Sampling technique and procedure

**Figure 1:**
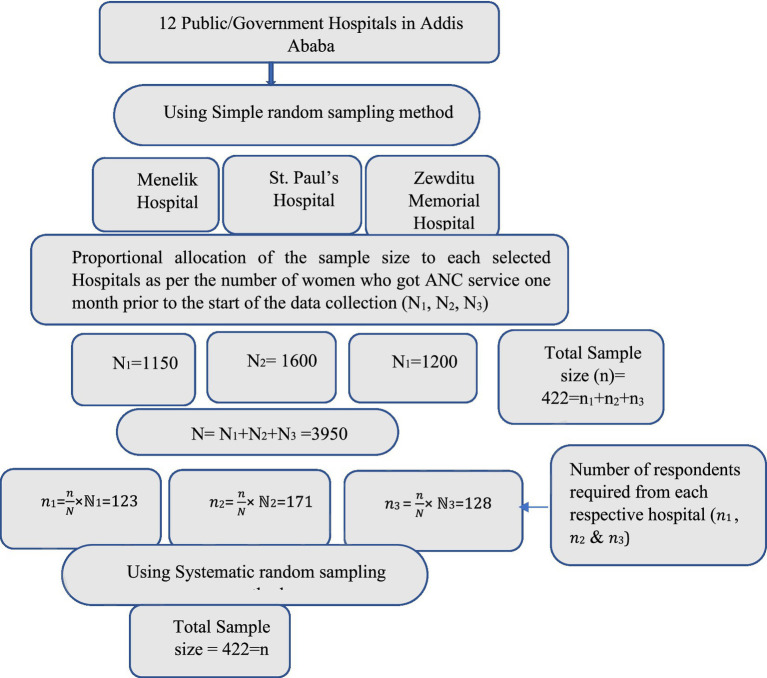
Sampling procedure of the study.

On the basis of previous hospital records, the total number of pregnant women who had ANC follow-up at each selected hospital (within a month before the start of data collection) was obtained. This was followed by a proportional allocation of the total sample size, based on the number of ANC attendees, in the respective hospitals. Using the ANC registry in the respective hospitals as a sampling frame, a systematic random sampling method was employed to select pregnant women who had ANC booking at the selected public hospital (Figure 1). Pregnant women receiving ANC services at each selected public hospital during the study period who met the inclusion criteria had a random chance of being approached. Consent was secured verbally and in written form before the start of the data collection.

#### Variables of the study

##### Dependent variable

Hemoglobin response to iron-folate supplementation (adequate or inadequate).

##### Independent variable

Sociodemographic factors (age, occupation, educational status, marital status, household size), Nutritional status, birth interval, parity, stage of pregnancy, timing of antenatal care booking, iron-folate supplementation (IFAS) adherence, duration of intake of IFAS, planned or unplanned pregnancy, and dietary diversity score.

### Operational definitions

Hemoglobin responses to IFA supplementation was categorized as either adequate or inadequate (9, 15). The criteria for these categories were as follows:

Adequate response: An increase in the hemoglobin value of at least 1 g/dL after a minimum of 1 month of supplementation, which is indicative of iron deficiency (16, 17).

Inadequate response: A change in the hemoglobin value of less than 1 g/dL after a minimum of 1 month of IFA supplementation. This response suggests the presence of functional iron deficiencies (9).

The reference range for classifying anemia, depicted below, was used (18). The severity of anemia was classified as follows: mild anemia (Hb: 10–10.9 g/dL), moderate anemia (Hb: 7–9.9 g/dL), and severe anemia (Hb < 7.0 g/dL) (5). Gestational ages were categorized into the first trimester (1–14 weeks), second trimester (15–28 weeks) and third trimester (29 and above) using WHO classification criteria. Pregnant women with a mid-upper arm circumference (MUAC) value of 23–33 cm were considered to have normal nutritional status; those with MUAC measurements <23 cm were categorized as undernourished, and those above 33 cm were categorized as obese (19, 20).

Compliance with the IFA supplement was assessed on the basis of pill count and self-reported methods. Women who took 70% or more of the IFA tablets, equivalent to taking at least 5 days a week throughout the study period, were considered adherent (21), using recording, self-reporting, pill counting and checking their cards. Otherwise, participants were considered non-adherent and excluded from the study.

**Figure 2:**
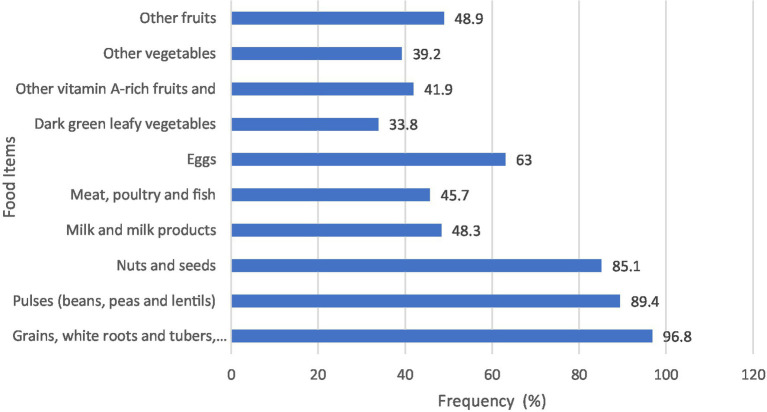
Dietary diversity scores, based on 24-h recall, of pregnant women attending antenatal care units in public hospitals in Addis Ababa.

Dietary intake was assessed via a food frequency questionnaire using the minimum dietary diversity for women (MDD-W), which was adopted by the Food and Agricultural Organization of the United Nations. The minimum dietary diversity for women (MDD-W) is a population-level indicator of diet diversity validated for women aged 15–49 years. It is a dichotomous indicator based on 10 food groups consumed locally (in this case, in Ethiopia) and is considered the standard for measuring population-level dietary diversity in women of reproductive age (22). Pregnant women who consumed five or more food items in the last 24 h, out of the 10 food groups, were considered to have adequate dietary diversity; otherwise, they were considered to have poor dietary diversity (Figure 2).

### Data collection procedures, tools and quality control

#### Data collection tools

A structured questionnaire was prepared in English language, adopted from previous research articles with minor revision (9, 22). The questionnaire was then translated into Amharic language to be filled by the respondents. The Amharic version was completed by the participants if they were able to read and write in the Amharic language; otherwise, the data collector completed the questionnaire by asking the respondents. The response was subsequently translated back into English. The questions explored and addressed the respondent’s sociodemographic profiles, data on parity/gravidity, intake of IFAS and dietary diversity.

#### Data collection procedure and quality control

Pregnant women who met the inclusion criteria were prescribed 60 mg of elemental iron plus 400 μg of folic acid oral tablets once daily if nonanemic and 120 mg of elemental iron plus 800 μg of folic acid once daily if anemic. The blood hemoglobin level of the participants was measured two times, i.e., at baseline, before the start of IFA supplementation and after the respondents were followed for at least 1 months. All the respondents were taking similar iron–folic acid tablets throughout the follow-up period.

A 5 mL venous blood sample was collected from respondents while they were seated comfortably, using iron-free heparinized test tubes. Professional nurses at each respective hospital, conducted the procedures. Hematological assessments were performed at both the baseline and the end point (after at least 4 weeks of IFAS supplementation). All standard precautions were strictly adhered to during and after blood collection. Hematological analysis was conducted via a Mindray Auto Hematology Analyzer (Mindray Biomedical Electronics Co. Ltd., China), with quality control measures rigorously followed according to the manufacturer’s guidelines.

Mid–upper arm circumference was measured via non-stretchable measuring tape according to the WHO recommendations to assess the participants’ nutritional status.

Data were collected by investigators assigned at each chosen hospital. To ensure the completeness, accuracy and consistency of data collection and for the investigators to have a common understanding of how to approach the participants, training was given before the start of data collection by the main investigator. A pretest was performed at Abebech Gobena MCH Hospital, Addis Ababa, on 10% of the sample to check for the accuracy of the responses, language clarity, and appropriateness of the tools. Following the pretest, some adjustments were performed, including some questions on the questionnaire being rephrased, data collectors being reoriented, and questionnaires being rearranged. On-site supervision was carried out by the principal investigator.

### Data processing and analysis

The completeness and consistency of the data were checked. The data were then entered into Epi-Data version 4.6 software. After the data were edited and coded, it was exported to SPSS version 27 software for further analysis. Hosmer and Lemeshow’s goodness-of-fit test was used to check the model fitness. All binary logistic regression assumptions were checked and fulfilled, and multicollinearity was studied using standard error and variance inflation factor. Bivariable binary logistic regression analysis was used to identify the variables that were related to adequate hemoglobin response to iron-folate supplementation. Variables that had a *p*-value of less than 0.25 in the bivariable binary logistic regression analysis were further entered into the multivariable binary logistic regression analysis. Variables having a *p*-value < 0.05, in the multivariable binary logistic regression analysis, were considered statistically significant. The adjusted odds ratio (AOR) with a 95% confidence interval (CI) was calculated to characterize the degree of the relationship between independent factors and adequate hemoglobin response to iron-folate supplementation.

### Ethical considerations

Ethical approval was obtained from research and ethical review committee of the department of Physiology, College of Health Sciences, AAU. Additional ethical approval was also obtained from Addis Ababa city health bureau and St. Paul’s Hospital Millennium Medical College with reference numbers A/A/12240/227 and Pm23/1024, respectively. Following receipt of the letter of approval, the investigator proceeded to the respective chosen hospitals to receive authorization letter for the collection of data from the respective hospitals. To maintain privacy and confidentiality, the respondent’s name was replaced by codes while the information was used only for the objectives of the study. The respondents were informed of the aim of the study, given the chance to withdraw from the study anytime they feel uncomfortable. Written informed consent was also obtained from each respondent.

## Results

### Sociodemographic characteristics of the study participants

Four hundred ten subjects participated in the study, with a response rate of 97%. The age of the study subjects ranged from 19 to 43 years, with a median age of 30 years and an IQR of 34–26 years. The majority of respondents (63.7%, *n* = 261) had completed secondary school, whereas 395 (96.3%) of the respondents were permanent urban residents. Unemployed respondents accounted for 274 (66.8%) of the participants. Most of the respondents were married (90.5%) (Table 1).

**Table 1:** Sociodemographic characteristics of pregnant women attending antenatal care units in selected public hospitals in Addis Ababa, Ethiopia.

Variables	Category	Frequency	Percentage
Age in years^*^	<20	4	1
	20–29	189	46.1
	30–39	209	51
	>40	8	2
Residency	Urban	395	96.3
	Rural	15	3.7
Occupation	Employed	136	33.2
	Unemployed	274	66.8
Marital status	Unmarried	39	9.5
	Married	371	90.5
Household size	1–5	384	93.7
	>5	26	6.3
Educational status	No formal education	11	2.7
	Attended primary school	45	11
	Attended secondary school	261	63.7
	Higher education	93	22.7

* Age category was adopted from a previous research article (10).

### Clinical characteristics of the study participants

Most of the participants (58.8%) were in their third trimester of pregnancy (i.e., at the end of the follow-up) with multiparity (54.4%), and more than half of the participants started ANC booking in the early first trimester. The majority of the respondents (80.5%) had a birth interval of more than 2 years. Two hundred eighty-five respondents reported that their pregnancy was planned and that more than half of the respondents had been taking IFAS for ≥2 months. The most common times for IFAS and coffee/tea intake were after meals (53.7 and 71%, respectively). Over half of the respondents (56.8%) had adequate MDD-W. Approximately three-fourths of the participants had MUAC measurements within the normal range of 23–33 cm (Table 2).

**Table 2:** Clinical characteristics of pregnant women attending antenatal care units in selected public hospitals in Addis Ababa (*n* = 410).

Variables	Category	Frequency	Percentage
Pregnancy stage	Second trimester	169	41.2
	Third trimester	241	58.8
Parity	≤1	84	20.5
	2–4	223	54.4
	≥5	103	25.1
Duration of IFAS	≥2 months	206	50.3
	1–2 months	204	49.7
IFA intake timing	After meal	220	53.7
	Before meal	126	30.7
	No time preference	64	15.6
Birth interval	<2 years	80	19.5
	≥2 years	330	80.5
MDD-W	Adequate	233	56.8
	Inadequate	177	43.2
Nutritional status	<23 cm	69	16.8
	23–33 cm	311	75.9
	>33 cm	30	7.3
ANC booking	Early first trimester	222	54.15
	Late first trimester	188	45.85
Coffee/tea intake	After meal	291	71
	Before meal	52	12.7
	No time preference	67	16.3
Pregnancy	Planned	285	69.5
	Unplanned	125	30.5

MDD-W, Minimum Dietary Diversity for Women; IFAS, Iron-folate supplementation, Early first trimester: <7 weeks after conception, late first trimester ≥7 weeks after conception.

### Hematological responses to IFA supplementation

An adequate Hb response, an increase in hemoglobin level by 1 g/dL and above, was found in 40.7% of the respondents who took IFA. Of the 161 women who had anemia, 91 of them overcame their anemia after supplementation, and 70 respondents continued to have anemia even after supplementation. Sixty-three participants (15.3%) who were anemic before IFAS initiation also remained anemic after supplementation. Eight pregnant women with normal baseline hemoglobin levels developed anemia despite intake of IFAS.

### Factors associated with the hemoglobin response to IFA supplementation

The variables that were entered into multivariable logistic regression analysis were marital status, planned or unplanned pregnancy, parity, MDD-W, duration of IFAS intake, the timing of ANC booking, experiencing side effects of IFAS and the number of tablet intakes per week (intermittent intake or continuously all days of the week for the duration of the supplementation) (Table 3).

**Table 3:** Multivariable binary logistic regression of factors associated with the Hb response to iron folate supplementation among pregnant women attending antenatal care units in Addis Ababa public hospitals.

Variables	Adequate response to IFAS *n* (%)	Inadequate response to IFAS *n* (%)	COR (95% CI)	AOR (95% CI)	*p* value
Marital status					
Married	158	213	2.5 (1.1–5.4)	1.8 (0.7–4.4)	0.23
Single	9	30	1	1	
Pregnancy					
Intended	135	150	2.6 (2.64–4.16)	1.6 (1.1–3.5)	0.15
Unintended	32	93	1	1	
Parity					
<2	48	36	2.3 (1.4–3.7)	2.4 (1.4–4.2)	0.003
≥2	119	207	1	1	
Timing of ANC booking					
≤7 Weeks	119	103	3.4 (2.2–5.13)	3.9 (2.4–6.6)	0.001
>7 weeks	48	140	1	1	
Duration of IFAS intake					
≥2 months	110	96	2.95 (1.96–4.5)	2.6 (1.6–4.2)	0.001
1–2 months	57	147	1	1	
Side effects from IFAS					
Yes	81	159	0.49 (0.3–0.7)	0.5 (0.3–0.8)	0.007
No	86	84	1	1	
Tablet intake per week					
>5 tabs	142	155	3.2 (1.96–5.30)	2.3 (1.3–4.1)	0.005
≤5 tabs	25	88	1	1	
MDD-W					
Adequate	147	30	2.55 (1.7–3.8)	3.4 (2.1–5.6)	0.001
Inadequate	96	137	1	1	

ANC, antenatal care; AOR, adjusted odds ratio; COR, crude odds ratio; CI, confidence interval; IFAS, iron folic acid supplementation; MDD-W, minimum dietary diversity for women.

Among all the variables included in the multivariable logistic regression analysis, MDD-W, the timing of ANC booking, the duration of IFAS intake, the average number of tablets consumed per week, parity and experiencing IFA side effects were significantly associated with the main outcome variable (adequate Hb response after supplementation) after adjustment for confounding factors (Table 3).

The respondents who took IFA tablets for more than 2 months were 2.6 times more likely to have adequate hemoglobin responses than respondents who took for a duration of 1–2 months [AOR = 2.6, 95% CI (1.6–4.2)]. Additionally, pregnant women who reported that they had side effects from IFA intake were less likely to have an adequate response to IFA supplementation than were those who did not experience side effects. (AOR = 0.5, 95% CI = 0.32–0.84). Furthermore, pregnant women who were adherent to IFAS were 2.3 times more likely to have an adequate hemoglobin response to iron folate supplementation than non-adherent respondents (AOR = 2.3, 95% CI = 1.28–4.1). This study also revealed that primiparous respondents were 2.4 times more likely to have an adequate Hb response to iron folate supplementation than multiparous respondents were [AOR = 2.4, 95% CI (1.4–4.2)] (Table 3).

Additionally, pregnant women with adequate minimum dietary diversity for women (MDD-W) were 3.4 times more likely to have an adequate hemoglobin response than those with inadequate MDD-W (AOR = 3.4, 95% CI = 2.12–5.56) (Figure 2). Additionally, pregnant women who booked for antenatal care at the health facility in the first 7 weeks of the first trimester were 3.9 times more likely to have an adequate hemoglobin response to iron-folate supplementation than respondents who booked in later gestational weeks (AOR = 3.9, AOR = 2.41–6.56).

## Discussion

Our study revealed that only 40.7% of pregnant women demonstrated an adequate hemoglobin response to iron-folate supplementation [95% CI: 35.9–45.5%]. This proportion is comparable to findings from Jordan (43.1%) (23), suggesting a similar effectiveness of the intervention across different populations. However, it is lower than the response rate reported in Mekelle, Ethiopia (48.5%) (9), and in a meta-analysis of five randomized controlled trials (17). The observed differences may be explained by geographical and physiological factors. Specifically, the higher altitude of Addis Ababa may demand greater hematological adaptation, which could reduce the responsiveness to iron-folate supplementation compared to women living in lower-altitude settings such as Mekelle.

Respondents with adequate dietary diversity scores were 3.4 times more likely to exhibit an adequate hemoglobin response to iron-folate supplementation compared to those with inadequate dietary diversity (AOR = 3.4, 95% CI: 2.12–5.6). This finding aligns with previous studies conducted in southern Ethiopia (24), North Shewa, Ethiopia (25), and Ghana (26), highlighting the positive impact of dietary diversity on hematological outcomes during pregnancy. However, it contrasts with another study in Ghana (27), which may be explained by the inclusion of a population with relatively better baseline nutritional status, potentially masking the effect of dietary diversity on hemoglobin response. Similarly, a multi-center cross-sectional study in Arba Minch, Ethiopia, reported no significant difference in minimum dietary diversity (MDD) scores between anemic and non-anemic participants (*p* = 0.12) (28), differing from the current study. Conversely, evidence from a prospective cohort study in Central Ethiopia demonstrated that low dietary diversity during pregnancy increased the risk of anemia at all stages, while achieving minimum women’s individual dietary diversity (WIDD) scores was associated with a reduced risk of anemia (29).

Additional community-based studies in Ethiopia have reported a higher risk of iron deficiency among women with low dietary diversity (aRR = 1.36; 95% CI = 1.07–1.72) (30) and a greater likelihood of co-occurring iron, folate, and vitamin A deficiencies in pregnant women with low consumption of diversified diets (AOR = 2.18; 95% CI = 1.35–3.51) (31). Supporting evidence also comes from studies conducted in Western China (32), Turkey (33) and Jordan (34), reinforcing the importance of achieving minimum dietary diversity for optimal micronutrient status and hematological health during pregnancy.

In this study, respondents who booked antenatal care in the first half of the first trimester, and consequently started IFAS earlier, had a significantly better hemoglobin response to iron-folate supplementation than those who booked in the latter half of the first trimester (AOR = 3.9, 95% CI: 2.41–6.56). Similar findings have been reported in studies conducted in Nigeria (35) and South Africa (36). Research indicates that iron supplementation during organogenesis (the first 3–8 weeks of gestation) may have teratogenic effects, suggesting that supplementation should be avoided during this period (37). However, in settings where anemia prevalence is high, pregnant women are recommended to begin iron-folate supplementation as early as possible after conception (5, 38).

Primiparous respondents were 2.4 times more likely to achieve an adequate hemoglobin response to iron-folate supplementation compared to multiparous women (AOR = 2.4, 95% CI: 1.4–4.2). This finding is in line with several previous studies (39, 40), which have similarly reported better hematological outcomes among first-time mothers. In contrast, some reports suggest a reduced risk of anemia with higher parity, possibly due to physiological adaptations or prior experience with antenatal care and supplementation (41, 42).

Our results are supported by prospective evidence showing that anemia and low serum ferritin levels are more prevalent in multiparous women than in nulliparous women (43). Comparable observations have also been made in studies conducted in Oman (44), Japan (3), Pakistan (45, 46), India (47), and Ghana (46) which consistently identify multiparity as a significant risk factor for suboptimal hemoglobin response during pregnancy.

Biologically, the increased susceptibility of multiparous women can be attributed to cumulative depletion of iron and other micronutrient stores over successive pregnancies (47). Additionally, accelerated plasma volume expansion in multiparous pregnancies may dilute hemoglobin concentration, limiting the effectiveness of iron-folate supplementation (48). These findings highlight the importance of tailored nutritional counseling, close monitoring of hemoglobin levels, and strategies to ensure sufficient micronutrient intake, particularly for multiparous women, to optimize hematological outcomes during pregnancy.

In this study, respondents who reported intake of the supplement for 2 months or more were 2.6 times more likely to achieve an adequate hemoglobin response to iron-folate supplementation compared to those who took it for only 1–2 months (AOR = 2.6, 95% CI: 1.6–4.2). This finding is consistent with a study conducted in Kenya (49) but not with a study from northern Ethiopia (9). The discrepancy between our findings and those from the study in northern Ethiopia may be due to differences in baseline nutritional status between the two populations, which can influence the efficacy of iron-folate supplementation.

Compared to those who took five or fewer tablets per week intermittently, respondents who consumed more than five iron-folate tablets per week on consecutive days were 2.3 times more likely to achieve an adequate change in hemoglobin levels (AOR = 2.3, 95% CI: 1.28–4.1). This result aligns with previous research (49). However, a randomized controlled trial (RCT) involving 200 participants found no significant differences in hemoglobin levels, serum ferritin, or reticulocyte counts between alternate-day oral iron supplementation and continuous daily supplementation (50, 51). Our findings also contradict previous studies that assessed the efficacy of daily versus intermittent iron intake in anemia prevention and reported inconclusive results (50, 52). Additionally, a randomized controlled trial suggested that daily iron supplementation increases serum hepcidin levels, reducing iron absorption (53). In contrast, studies have shown that alternate-day dosing optimizes iron absorption and may be a preferable regimen (54).

Respondents who reported experiencing at least one known side effects such as nausea, constipation, gastrointestinal discomfort, and metallic taste from iron-folate supplementation had a poorer hemoglobin response compared to those who did not report side effects. The side effects reported by participants included gastrointestinal discomfort, nausea, constipation, and vomiting. Pregnant women who experienced at least one of the mentioned side effects were approximately 50% less likely to achieve an adequate hemoglobin response (AOR = 0.5, 95% CI: 0.32–0.84) (Table 3). This could be due to inconsistent adherence, as side effects may lead to reduced supplement intake. Side effects may have led to inconsistent adherence to the supplementation regimen, thereby reducing its effectiveness. This finding aligns with previous studies that suggest side effects are a common barrier to iron-folate supplementation adherence, particularly with daily dosing regimens. Studies also suggest that intermittent regimens of iron-folate supplementation result in fewer side effects than daily supplementation thereby increasing adherence to iron-folate supplementation and overall effectiveness of the intervention (55).

## Conclusion and recommendations

### Conclusion

In this study, iron-folate supplementation was found to be ineffective in preventing anemia in a substantial proportion (17%) of women and resulted in an inadequate hemoglobin response in nearly 60% of the participants. Dietary diversity, an earlier timing of ANC booking, a duration of IFAS intake of more than 2 months, compliance with iron folate supplementation, and primiparity were found to be significantly associated with adequate hemoglobin response to iron-folate supplementation.

### Recommendations

Given the study findings, it is recommended to implement strategies that promote dietary diversity, encourage early antenatal care booking, ensure adequate duration of iron-folate supplementation, and monitor adherence to supplementation regimens to improve the efficacy of iron-folate supplementation in preventing anemia during pregnancy.

### Strength and limitations of the study

#### Strength of the study

This study provides primary, context-specific evidence on determinants of hemoglobin response to iron-folate supplementation among pregnant women, with a focus on dietary diversity, timing of ANC visit, and parity—factors that are often underexplored in similar settings. The use of a robust study design and appropriate statistical methods, including adjustment for potential confounders, enhances the validity of the findings. Third, integrating multiple biochemical and dietary measures allows for a comprehensive understanding of nutritional and hematological status.

We believe this study contribute to knowledge by highlighting the critical role of adequate dietary diversity, timing of ANC visit, and parity in optimizing hemoglobin response, emphasizing the need for tailored nutritional counseling and targeted interventions for multiparous women and those with limited dietary diversity. These findings can inform public health strategies aimed at improving maternal nutrition and reducing anemia prevalence in similar low-resource settings.

#### Limitations of the study

In this study, additional tests that could provide a more accurate assessment of iron levels such as measuring serum iron, total iron-binding capacity (TIBC), and ferritin were not performed due to cost limitations. Also, when evaluating respondents’ dietary diversity, recall bias might have affected the accuracy of the information reported by the participants. Finally, due to time constraints, a third hemoglobin measurement, could not be conducted.

## Data Availability

The raw data supporting the conclusions of this article will be made available by the authors without undue reservation.

## Glossary

ANC: Ante Natal Care
AOR: Adjusted Odds Ratio
CSA: Central Statistical Agency
CI: Confidence Interval
EDHS: Ethiopia Demography and Health Survey
EPHI: Ethiopian Public Health Institute
FAO: Food and Agricultural Organization of the United Nations
FPN: Ferroportin
FMOH-E: Federal Ministry of Health, Ethiopia
GA: Gestational Age
Hb: Hemoglobin
IDA: Iron Deficiency Anemia
IFAS: Iron-Folic Acid Supplementation
MDD-W: Minimum Dietary Diversity for Women
OR: Odds Ratio
RCT: Randomized Controlled Trials
SPHMMC: St. Paul’s Millenium Medical College
SPSS: Statistical Package for Social Sciences
TIBC: Total Iron Binding Capacity
WHO: World Health Organization
